# Linear growth following complicated severe malnutrition: 1-year follow-up cohort of Kenyan children

**DOI:** 10.1136/archdischild-2018-315641

**Published:** 2018-09-28

**Authors:** Moses M Ngari, Per Ole Iversen, Johnstone Thitiri, Laura Mwalekwa, Molline Timbwa, Greg W Fegan, James Alexander Berkley

**Affiliations:** 1 KEMRI/Wellcome Trust Research Programme, Kilifi, Kenya; 2 Childhood Acute Illness and Nutrition (CHAIN) Network, Nairobi, Kenya; 3 Department of Nutrition, IBM, University of Oslo, Oslo, Norway; 4 Department of Hematology, Oslo University Hospital, Oslo, Norway; 5 Division of Human Nutrition, Stellenbosch University, Tygerberg, South Africa; 6 Swansea Trials Unit, Swansea University Medical School, Swansea, UK; 7 Centre for Tropical Medicine and Global Health, University of Oxford, Oxford, UK

**Keywords:** malnutrition, undernutrition, stunting, growth, height

## Abstract

**Background:**

Stunting is the most common manifestation of childhood undernutrition worldwide. Children presenting with severe acute malnutrition (SAM) are often also severely stunted. We evaluated linear growth and its determinants after medically complicated SAM.

**Methods:**

We performed secondary analysis of clinical trial data **(**NCT00934492) from HIV-uninfected Kenyan children aged 2–59 months hospitalised with SAM. Outcome was change in height/length-for-age z-score (HAZ) between enrolment and 12 months later. Exposures were demographic, clinical, anthropometric characteristics and illness episodes during follow-up.

**Results:**

Among 1169 children with HAZ values at month 12 (66% of those in original trial), median (IQR) age 11 (7–17) months and mean (SD) HAZ −2.87 (1.6) at enrolment, there was no change in mean HAZ between enrolment and month 12: −0.006Z (95% CI −0.07 to 0.05Z). While 262 (23%) children experienced minimal HAZ change (within ±0.25 HAZ), 472 (40%) lost >0.25 and 435 (37%) gained >0.25 HAZ. After adjusting for regression to the mean, inpatient or outpatient episodes of diarrhoea and inpatient severe pneumonia during follow-up were associated with HAZ loss. Premature birth and not being cared by the biological parent were associated with HAZ gain. Increases in mid-upper arm circumference and weight-for-age were associated with HAZ gain and protected against HAZ loss. Increase in weight-for-height was not associated with HAZ gain but protected against HAZ loss. No threshold of weight gain preceding linear catch-up growth was observed.

**Conclusions:**

Interventions to improve dietary quality and prevent illness over a longer period may provide opportunities to improve linear growth.

**What is already known on this topic?**

- Children presenting with severe acute malnutrition (SAM) are usually also severely stunted suggesting that their malnutrition is often not only acute.
- Several studies have reported minimal average height/length-for-age z-score (HAZ) gain during recovery from SAM despite intense nutritional support and weight gain.
- A threshold of wasting recovery before linear catch-up growth has previously been proposed.

**What this study adds?**

- Despite an insignificant change in mean HAZ subgroups of children gained or lost HAZ beyond that predicted by regression to the mean.
- No threshold of wasting recovery preceding linear catch-up growth was observed.
- More active prevention and treatment of illness following SAM treatment may provide opportunities to improve linear growth.

## Introduction

Stunting (defined as height/length-for-age z-score (HAZ) ≤2 using WHO 2006 growth reference standards) is the most prevalent form of undernutrition: 151 million children under 5 years old were stunted in 2017.^1–4^ A Sustainable Development Goal has been set by the United Nations to reduce the proportion of children who are stunted worldwide by 40% by 2025.^5^ More than 90% of stunted children live in Africa and Asia.^1^ In Kenya, 26% of under-5s were stunted in 2014,^6^ with wide subnational variation, ranging from 15% in central Kenya to 46% on the coast.^6 7^ Stunting is considered to be a result of chronic malnutrition, exacerbated by frequent infections, especially diarrhoea.^8 9^ In resource-poor countries, it is estimated that approximately a fifth of stunting occurs in utero and continues up to 2 years of age.^10^ Being stunted is associated with reduced cognitive development and adult productivity, adverse maternal reproductive outcomes and risks of non-communicable diseases in adulthood.^3 11–13^ Despite being globally recognised as the most frequent form of undernutrition, stunting is often overlooked in the clinic because length/height is not routinely measured in health facilities in affected countries, some communities consider short stature as ‘normal’, and specific effective interventions are lacking.^3^

An overview of published clinical trials among children with severe acute malnutrition (SAM) reveals that they are also usually severely stunted, thus their malnutrition is not only acute (listed in online supplementary table 1).^14 15^ The main target of treatment and nutritional rehabilitation of complicated SAM is to increase body weight and/or mid-upper arm circumference (MUAC).^16–18^ It might therefore be expected that such intervention would also impact on linear growth. However, clinical trials of treatment for SAM in Asia and Africa demonstrate that children with SAM have little or no change in average HAZ following treatment (online supplementary table 1).^19–23^ In one study of 369 predominantly oedematous Jamaican children recovering from severe malnutrition, Walker *et al* observed that in a subgroup who were more severely stunted and mostly non-oedematous, weight gain preceded catch-up in linear growth and proposed that reaching a threshold of weight-for-height/length z-scores (WHZ) of −1.3 was required before linear catch-up growth could begin.^24^

10.1136/archdischild-2018-315641.supp1 Supplementary data

We therefore evaluated linear growth and its associated factors among HIV-uninfected children enrolled in a multicentre randomised clinical trial during recovery from complicated SAM and followed up for 12 months.^23^

## Patients and methods

### Study design and setting

We performed a secondary analysis of data from a double-blinded, randomised placebo-controlled trial (ClinicalTrials.gov NCT00934492).^23^ The original trial was designed to determine the efficacy of daily co-trimoxazole prophylaxis in reducing postdischarge mortality among children with complicated SAM. The intervention had no overall effect on linear growth.^23^

The trial recruited children from paediatric wards of four Kenyan hospitals while receiving treatment for complicated SAM: Mbagathi County Hospital (Nairobi), Coast General Hospital (Mombasa), Kilifi County Hospital and Malindi Subcounty Hospital.

### Study population

The original trial recruited children aged 2–59 months, hospitalised with complicated SAM defined as: MUAC <11 cm for children <6 months, MUAC <11.5 cm for children 6–59 months, or oedema at any age.^23^ Children were excluded if they had positive HIV rapid antibody test, because they were already recommended to receive daily co-trimoxazole prophylaxis, or had known hypersensitivity to co-trimoxazole. Children were enrolled into the trial after the initial ‘stabilization’ phase of SAM inpatient treatment and caregiver consent was provided.^18^ Participants were scheduled to attend clinic visits monthly after study enrolment up to month 6 and thereafter, every 2 months up to month 12, giving a total of 1-year follow-up.

### Study outcomes

The main study outcome was change in HAZ between study enrolment and month 12. The exposures of interest were age, gender, recruitment site, gain in weight-for-age z-score (WAZ), WHZ and MUAC, clinical variables at enrolment, randomisation arm and illness episodes occurring during follow-up.

### Data sources and measurements

At enrolment, clinical history, disease signs and sociodemographic data were collected. During enrolment and at every scheduled visit, length (or height for children ≥24 months old), weight and MUAC were measured and documented.^23^

During follow-up, hospital readmissions and illness episodes treated as an outpatient were prospectively documented. Study staff visited the homes of children failing to attend scheduled visits. During these home visits, vital status was assessed and MUAC measured, but weight and height were not measured because of the measurement equipment required.

### Study size

We used data from all (n=1169) participants who (1) survived, (2) remained in the 1-year follow-up, and (3) had their HAZ measured at study end.

### Statistical methods

All analyses were conducted using Stata V.15.1 (StataCorp, College Station, TX, USA). HAZ, WHZ and WAZ were calculated using the 2006 WHO growth reference. Change in HAZ between enrolment and month 12 was categorised into three groups: minimal change in HAZ of −0.25 to 0.25; loss of HAZ >0.25; and gain in HAZ >0.25 according to Walker and Golden.^24^ We hypothesised that gain or loss of HAZ may be associated with different baseline factors and these were thus analysed as separate outcomes using the minimal change group as the reference. To evaluate the effect of regression to the mean (RTM), gain or loss of HAZ was adjusted for the difference between individuals’ HAZ values and the group mean HAZ value at enrolment.^25^ We used backwards stepwise multinomial logistic regression to determine the features associated with either loss or gain, retaining variables with p<0.1, and reported adjusted coefficients transformed to relative risk ratios with p<0.05 in the final model. We also performed a sensitivity analysis to explore the factors associated with HAZ loss or gain from baseline to month 12 among stunted children at baseline only. To determine the extent to which overall changes in weight, WAZ, WHZ and MUAC were associated with HAZ changes during follow-up, we calculated the Spearman’s rank correlation coefficient after excluding children with oedema at enrolment, and examined other factors associated with change in HAZ using multinomial logistic regression.

To look for evidence that a curve indicating inflection or threshold characterised the relationship between changes in HAZ and changes in weight, WAZ, WHZ and MUAC rather than a straightforward linear relationship, we performed multivariable fractional polynomial regression (MFPR) adjusted for age, gender, study site and randomisation arm. Regression models based on fractional polynomial curves (powers of −2, −1, −0.5, 0, 0.5, 1, 2, 3) were compared with a linear model by testing for difference in deviance using the STATA *mfp* command. The fractional polynomial term was retained if there was significant difference in deviance from the linear model.^26^

To test the threshold proposed by Walker and Golden, we compared linear growth among children, including those with oedema at enrolment, above and below the threshold of WHZ −1.3 or more after 1 month, using a Wilcoxon rank-sum test.^24^

## Results

The original trial enrolled 1778 children. We excluded 609 (34%) children; (257 (14%) died, 56 (3.1%) were lost to follow-up, 36 (2.0%) voluntarily withdrew and 260 (15%) had no HAZ measured at month 12). Thus, 1169 children were included in this analysis, median (IQR) age at enrolment was 11 (7 – 17) months, and 565 (48%) were girls (table 1). Mean (SD) HAZ at enrolment was −2.87 (1.6). Overall, 358 (31%), 290 (25%) and 521 (44%) were not stunted, moderately and severely stunted, respectively (online supplementary table 2 and online supplementary figure 1). Baseline stunting was associated with older age, female sex, recruitment hospital, reported low birth weight, presence of oedema and lower MUAC (online supplementary tables 3 and 4). Children who were excluded had similar enrolment characteristics, but a higher frequency of postdischarge illness episodes (under follow-up events in table 1).

**Table 1:** Study participants’ profile at time of enrolment and follow-up

	All participants in primary trial (n=1778)	Participants assessed at month 12 and included in the secondary analysis (n=1169)
Demographics at study enrolment		
Age in months, median (IQR)	11 (7–16)	11 (7–17)
Sex, female	875 (49)	565 (48)
Born prematurely*	221 (12)	141 (12)
Born underweight†	362 (20)	229 (20)
Main caregiver-biological mother	1661 (93)	1088 (93)
Randomised to co-trimoxazole prophylaxis	887 (50)	597 (51)
Currently breast feeding	1092 (61)	712 (61)
Recruitment hospital		
Kilifi County Hospital	151 (8.5)	114 (9.8)
Coast General Hospital	849 (48)	542 (46)
Malindi Subcounty Hospital	271 (15)	202 (17)
Mbagathi County Hospital	507 (29)	311 (27)
Nutritional status at study enrolment		
Oedema	300 (17)	210 (18)
MUAC (cm), mean±SD	10.6±1.1	10.6±1.0
HAZ, mean±SD	−2.87±1.7	−2.87±1.6
WAZ, mean±SD	−3.99±1.0	−3.96±1.0
WHZ, mean±SD	−3.34±1.3	−3.32±1.2
Haemoglobin (g/dL), mean±SD	9.9±2.2	9.8±2.3
Index admission diagnosis		
Index admission with diarrhoea	1021 (57)	679 (58)
Index admission with severe pneumonia	656 (37)	404 (35)
Index admission with tuberculosis	67 (3.8)	45 (3.9)
Index admission with clinical signs of rickets	230 (13)	139 (12)
Index admission with other comorbidities‡	97 (5.5)	66 (5.7)
Follow-up events		
Outpatient treatment for diarrhoea	653 (37)	329 (28)
Outpatient treatment for pneumonia	716 (40)	275 (24)
Outpatient treatment for another diagnosis§	547 (31)	387 (33)
Hospital readmission for diarrhoea	218 (12)	102 (8.7)
Hospital readmission for severe pneumonia	308 (17)	159 (14)
Hospital readmission for another diagnosis§	236 (13)	96 (8.2)
Died	257 (14)	–
Withdrawn or loss to follow-up	92 (5.2)	–

The results are n (%) except when specified.

WAZ and WHZ exclude children with oedema. Outpatient treatment comprised any treatment offered to the participant either during scheduled or unscheduled follow-up visits.

*Gestational age <37 weeks.

†Birth weight <2500 g.

‡Comorbidity assessed at study enrolment including 5 sickle cell, 15 heart disease, 38 cerebral palsy, 3 epilepsy and 5 children with both cerebral palsy and epilepsy.

§Diagnoses of malaria, tuberculosis, sepsis, meningitis, measles, anaemia, and urinary tract infection and skin/soft tissue infection.

HAZ, height/length-for-age z-score; MUAC, mid-upper arm circumference; WAZ, weight-for-age z-score; WHZ, weight-for-height/length z-score.

The largest monthly change in HAZ was from enrolment to month 1, a drop (SD) of 0.20 (0.5) z-scores (online supplementary table 3, online supplementary figures 2 and 3). After month 1, mean (SD) HAZ rose to −2.94 (1.4) at month 4 and thereafter plateaued to month 12. In contrast, during the first month, WAZ, WHZ and MUAC (cm) had their largest gains (online supplementary table 5 and online supplementary figure 2).

**Figure 1:**
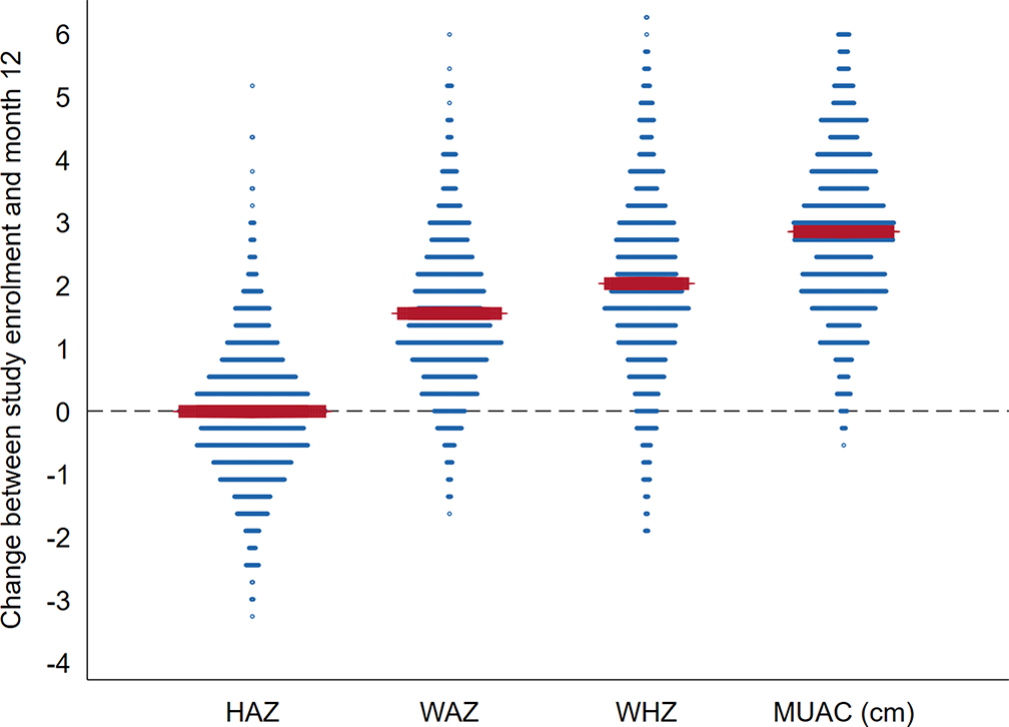
Dot plot of change between study enrolment and month 12 in HAZ, WHZ, WAZ and MUAC (cm). The dashed line (Y=0) indicates no change; the horizontal red bars are the respective anthropometry mean change. HAZ, height/length-for-age z-score; MUAC, mid-upper arm circumference; WAZ, weight-for-age z-score; WHZ, weight-for-height/length z-score.

At month 12, the mean (SD) HAZ was −2.88 (1.4), a change (SD) of −0.006 (1.1) since enrolment (figure 1 and online supplementary table 5). WAZ, WHZ and MUAC at month 12 had increased (SD) since enrolment by 1.46 (1.2), 1.94 (1.5) and 2.83 (1.4) cm, respectively (figure 1, online supplementary table 5 and online supplementary figure 2).

Of the 1169 children, 262 (23%) had minimal change in HAZ from enrolment to month 12, while 472 (40%) lost >0.25 and 435 (37%) gained >0.25 z-scores (online supplementary table 6). Most children (744/1169; 64%) were in the same HAZ category (severely, moderately or not stunted) at month 12 as they had been at study enrolment (online supplementary table 2 and online supplementary figure 1).

### Features associated with changes in HAZ

The youngest and oldest children had the largest change in HAZ, giving age a skewed U-shaped association (p<0.001 compared with a linear fit) (online supplementary figure 4). Age <12 months was associated with losing HAZ in the univariate analysis (online supplementary table 7).

In the multivariate analyses, losing HAZ was associated with postdischarge outpatient treatment for diarrhoea, hospital readmission for diarrhoea and for severe pneumonia, and randomisation to co-trimoxazole prophylaxis after adjusting for RTM (table 2). Gain in HAZ was associated with the caregiver not being the child’s biological mother, reported preterm birth and randomisation to co-trimoxazole after adjusting for RTM (table 2). Children born preterm had lower HAZ at enrolment than other participants (p<0.001), and their HAZ increased (SD) during the 1-year period to −3.04 (1.3), which was not significantly different from the other children (−2.85 (1.4)) (p=0.06) (online supplementary figure 5). In a sensitivity analysis, the factors associated with HAZ gain or loss among children who were already stunted at enrolment were found to be similar to those of the overall cohort (online supplementary table 8).

**Table 2:** Factors associated with loss or gain of HAZ during the 1-year follow-up

Demographics at study enrolment	Lost at least 0.25 HAZ*			Gained at least 0.25 HAZ*		
	Adjusted RRR	95% CI	P values	Adjusted RRR	95% CI	P values
Main caregiver not the biological mother	–	–	–	2.42	1.19 to 4.92	0.02
Randomised to co-trimoxazole prophylaxis	0.70	0.51 to 0.96	0.03	0.72	0.52 to 0.99	0.05
Born prematurely†	–	–	–	1.97	1.18 to 3.28	0.01
Follow-up illness events						
Outpatient treatment for diarrhoea	1.68	1.15 to 2.44	0.007	–	–	–
Readmission to hospital for diarrhoea	1.91	1.02 to 3.55	0.04	–	–	–
Readmission to hospital for severe pneumonia	1.90	1.12 to 3.22	0.02	–	–	–

*Compared with children with minimal change in HAZ (±0.25Z). HAZ difference is the difference between HAZ at month 12 and at study enrolment.

†Gestational age <37 weeks. Relative risk ratios are computed using multinomial logistic regression with minimal HAZ change (−0.25 to 0.25Z) as the reference and adjusted for regression to the mean. All the factors examined in the multivariate multinomial logistic regression are reported on univariate analysis (online supplementary table 5). Here only factors with a p value <0.05 are presented; p values are from multivariable multinomial logistic regression.

HAZ, height/length-for-age z-score; RRR, relative risk ratio.

**Figure 2:**
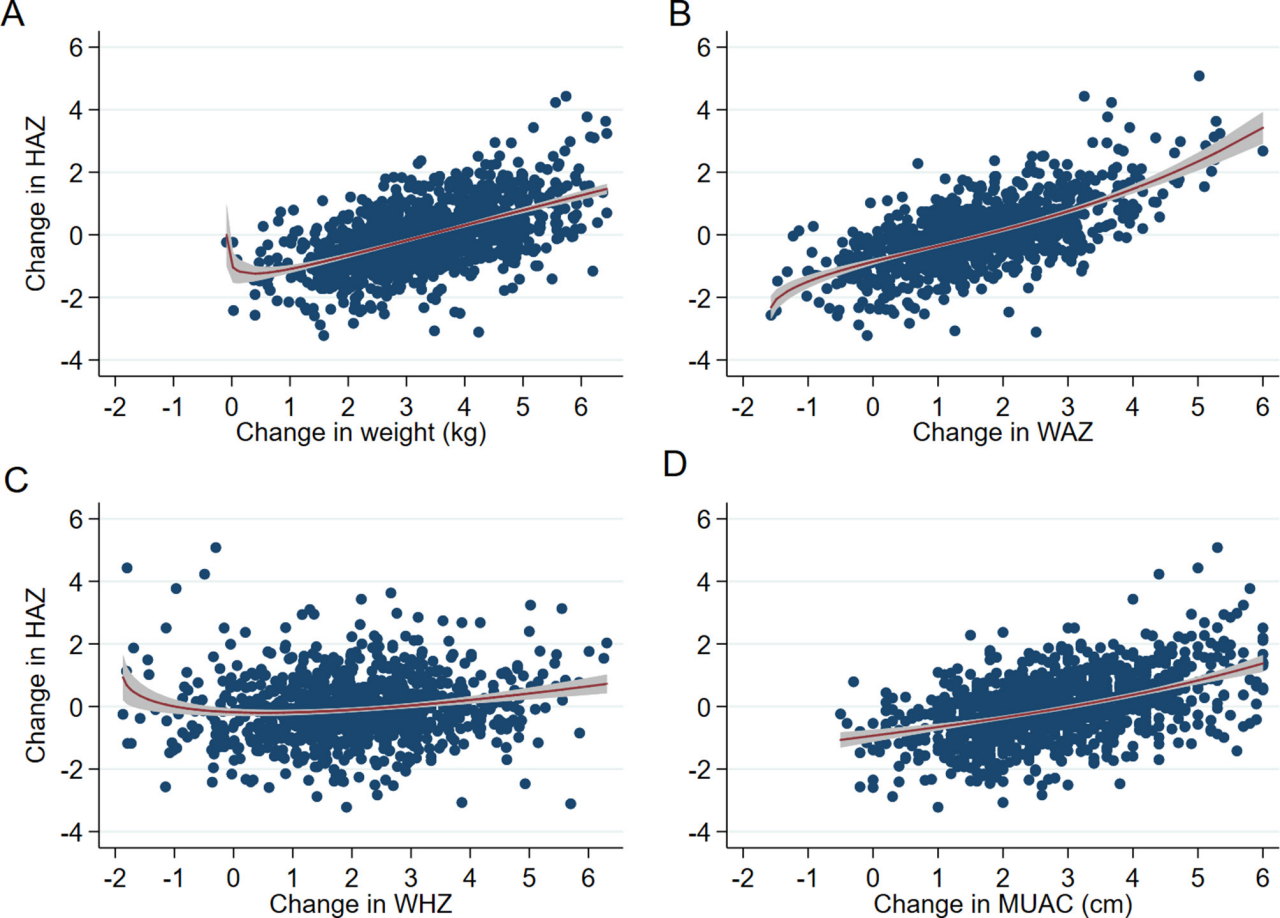
Scatter plots of change in HAZ with: (A) change in weight (kg) (p=0.25), (B) changes in WAZ (p=0.11), (C) change in WHZ (p=0.07), and (D) change in MUAC (cm) (p=0.24) with fitted fractional polynomial curves (95% CI). P values are from comparisons of model deviance between multiple fractional polynomial regression and linear regression models. HAZ, height/length-for-age z-score; MUAC, mid-upper arm circumference; WAZ, weight-for-age z-score; WHZ, weight-for-height/length z-score.

Changes in weight, WAZ and MUAC between enrolment and month 12 had strong positive correlation with absolute change in HAZ, while change in WHZ had weak correlation (table 3). Overall, there was no evidence from the MFPR analyses of an inflection in the association between change in HAZ up to month 12 and change in weight, WAZ, WHZ or MUAC (figure 2).

**Table 3:** Multivariable analysis of association between HAZ change and changes in weight, WAZ, WHZ and MUAC during 1-year follow-up

Change between study enrolment and month 12	Correlation coefficient†	Lost at least 0.25 HAZ*			Gained at least 0.25 HAZ*		
		Adjusted RRR‡	95% CI	P values	Adjusted RRR†	95% CI	P values
Change in weight per kg	0.55	0.46	0.39 to 0.56	<0.001	2.15	1.82 to 2.54	<0.001
Change in WAZ per z-score	0.61	0.44	0.35 to 0.55	<0.001	2.19	1.77 to 2.71	<0.001
Change in WHZ per z-score	0.15	0.80	0.69 to 0.92	0.002	1.10	0.95 to 1.27	0.19
Change in MUAC per cm	0.46	0.69	0.60 to 0.80	<0.001	1.46	1.28 to 1.68	<0.001

*Compared with children with minimal change in HAZ (±0.25Z).

†Spearman’s rank correlation coefficient.

‡Adjusted for co-trimoxazole randomisation arm, child carer, born premature (<37 weeks), height-for-age at study enrolment, outpatient treatment for diarrhoea and readmission during follow-up for diarrhoea. Relative risk ratios are computed using multinomial logistic regression with minimal HAZ change (−0.25 to 0.25Z) as the reference. P values from multivariable multinomial logistic regression.

HAZ, height/length-for-age z-score; MUAC, mid-upper arm circumference; RRR, relative risk ratio; WAZ, weight-for-age z-score; WHZ, weight-for-height/length z-score.

**Figure 3:**
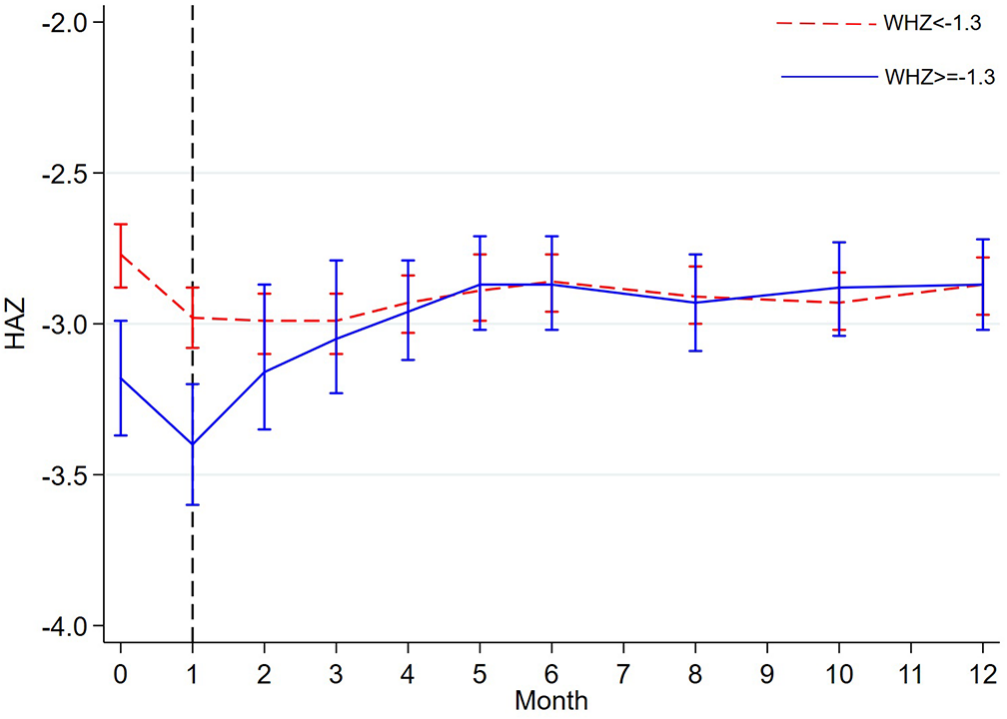
HAZ trajectory between participants below and above WHZ −1.3 at month 1 of follow-up. The dashed vertical line show time point when the WHZ used to group the participants was taken (month 1); anthropometry was not collected at months 7, 9 and 11; the plotted data are means and 95% CI. HAZ, height/length-for-age z-score; WHZ, weight-for-height/length z-score.

At month 1,275 (24%) children were above the threshold proposed by Walker and Golden^24^ of WHZ −1.3. These children had fewer baseline comorbidities, were less wasted and more stunted at enrolment than children below the threshold (online supplementary table 9). Between enrolment and month 1, children lost approximately 0.2 HAZ irrespective of reaching WHZ −1.3 or not (p=0.40) (figure 3 and online supplementary table 9). Linear growth between months 1 and 3 was greater in children who were above WHZ −1.3 than children below this threshold at month 1 (p<0.001). However, from month 3 to month 12 there were no differences in absolute values or changes in HAZ between these two groups (p=0.23) (figure 3 and online supplementary table 9).

## Discussion

Despite inpatient, medical and nutritional treatment for complicated SAM and subsequent outpatient therapeutic feeding, active follow-up to 1 year with access to extra care and referral for specialist services where needed by the study team, there was no significant average increase in HAZ. This was despite rapid weight and MUAC gain.

Previous trials that monitored children recovering from SAM in Africa have similarly demonstrated catch-up in weight but not in linear growth.^20–22 27^ In a community-based study in Nepal, wasted children gained weight but not height, suggesting wasted children experience weight growth at the expense of growing length.^28^ However, severely malnourished children in Jamaica experienced increased growth in both weight and height, although linear growth tended to occur later than weight growth.^29 30^

Overall, an increase in WHZ was not associated with positive linear growth in our study and there was no evidence of a threshold WAZ or WHZ gain required for a linear growth catch-up. When we examined a previously proposed threshold of wasting^24^ in relation to linear growth, we found a short period of accelerated linear growth among a subset of children with very different baseline characteristics, suggesting that early linear growth response predominantly depends on baseline status, such as the presence or absence of comorbidities.

Whereas having episodes of diarrhoea during follow-up was associated with linear growth deficit, length was not gained by not having diarrhoea. This concurs with results from a pooled analysis of data from seven studies in the general community that found diarrhoea was associated with long-term decrease in linear growth.^31^ Previous studies have also reported other infectious diseases such as malaria and respiratory illnesses may be associated with linear growth faltering.^14^ We found an association with hospital admission for severe pneumonia, but the occurrence of malaria was too rare to be included in our analyses separately. However, in a multicountry birth cohort in low/middle-income countries, while diarrhoea was not a risk factor for poor linear growth, but presence of higher enteropathogen load in non-diarrhoea stools was associated with poor length growth.^32 33^

Although not examined in this study, environmental enteric dysfunction (EED) common in resource-poor countries has been suggested as additional factor impacting on linear growth.^34^ EED is characterised by systematic inflammation of the small intestine, which is more frequent among children with SAM and likely to increase metabolic demands, altered nutrient processing by the microbiota and absorption, thereby affecting linear growth.^35 36^ Providing 81 Jamaican malnourished children with metronidazole to treat small intestinal bacterial overgrowth in addition to a high energy nutritional supplement improved both linear and weight growth compared with the supplement only.^37^ Metronidazole is now being tested in a large (n=2000) multicentre clinical trial among children with SAM (ISRCTN18051843).

The parent trial intervention, co-trimoxazole prophylaxis had a stabilising effect on HAZ, reducing both gain and loss. The mechanism for this is unclear but may reflect potential multiple effects of co-trimoxazole on infection, as well as on the gut microbiota involved in nutrient processing and absorption. Similarly, co-trimoxazole was associated with increased diarrhoea in the original trial, but reduced malaria, skin and urinary tract infections.^23^

Unlike findings from previous studies, after adjusting for other factors, age was not independently associated with HAZ changes.^38–40^ Children born prematurely had greater linear growth than their term peers in this study, reaching a similar HAZ to other children after 12 months of follow-up. However, typically preterm neonates may not fully catch up in length with full-term infants.^41^ Children not cared by their biological parents were mostly under the care of children’s homes where nutritional and emotional care may have promoted linear growth (sometimes this was initiated at the index admission by the hospitals’ social services when absence of a home carer was identified).

Pooled  and meta-analyses of numerous interventions to improve linear growth have demonstrated marginal efficacy.^34 42^ Bhutta *et al* predicted that implementing all the evidence-based nutritional interventions would only reduce stunting by 20% globally.^42^ However, a trial in Ecuador has recently demonstrated that introduction of daily eggs during the weaning period (6–9 months) reduced stunting by 47%.^43^ These results are encouraging, suggesting that protein quality over a prolonged period may be important and should be tested in different settings, including following treatment for SAM.

### Strengths and limitations

We systematically followed children for 1 year after inpatient treatment for complicated SAM, a longer period than most SAM studies.^19 22 24^ Being a clinical trial meant a higher level of clinical care and opportunities for linear growth gain that may not exist in routine care. We adjusted for the study intervention allocation in the multivariable regression analysis. A limitation of this study was selection bias because we could only analyse data from participants with length measurement at month 12. Although excluded children had similar baseline characteristics, those without a month 12 measurement because of death, withdrawal or not physically attending this visit may have been more likely to have linear growth deficit than included children because they were more often ill during follow-up (under follow-up events in table 1).

## Conclusion

Overall, intensive treatment and nutritional rehabilitation did not resolve stunting among children with SAM. Although reaching a previously proposed threshold of absolute WHZ of −1.3 was associated with increased short-term linear growth, this was seen in a subset of children who were less wasted, more stunted and with less comorbidity at enrolment. Interventions that prevent illness and improve dietary quality over a longer period may provide opportunities to improve linear growth following SAM.

## References

[1] Black RE, Victora CG, Walker SP, et al Maternal and child undernutrition and overweight in low-income and middle-income countries. Lancet 2013;382:427–51. 10.1016/S0140-6736(13)60937-X 23746772

[2] Prendergast AJ, Humphrey JH The stunting syndrome in developing countries. Paediatr Int Child Health 2014;34:250–65. 10.1179/2046905514Y.0000000158 25310000 PMC4232245

[3] de Onis M, Branca F Childhood stunting: a global perspective. Matern Child Nutr 2016;12:12–26. 10.1111/mcn.12231 27187907 PMC5084763

[4] United Nations Children’s Fund (UNICEF), World Health Organization (WHO), World Bank. Levels and trends in child malnutrition: key findings of the 2018 edition of the joint child malnutrition estimates. New York, Geneva, Washington, DC: UNICEF, WHO, The World Bank, 2018.

[5] Shekar M, Kakietek J, D’Alimonte MR, et al Reaching the global target to reduce stunting: an investment framework. Health Policy Plan 2017;32:657–68. 10.1093/heapol/czw184 28453717 PMC5406759

[6] Kenya National Bureau of Statistics (KNBS). Kenya Demographic and Health Survey 2014. Nairobi: KNBS, 2015.

[7] Kimani-Murage EW, Muthuri SK, Oti SO, et al Evidence of a double burden of malnutrition in urban poor settings in Nairobi, Kenya. PLoS One 2015;10:e0129943 10.1371/journal.pone.0129943 26098561 PMC4476587

[8] Scrimshaw NS, Taylor CE, Gordon JE Interactions of nutrition and infection. Monogr Ser World Health Organ 1968;57:3–329. 4976616

[9] Talbert A, Thuo N, Karisa J, et al Diarrhoea complicating severe acute malnutrition in Kenyan children: a prospective descriptive study of risk factors and outcome. PLoS One 2012;7:e38321 10.1371/journal.pone.0038321 22675542 PMC3366921

[10] Christian P, Lee SE, Donahue Angel M, et al Risk of childhood undernutrition related to small-for-gestational age and preterm birth in low- and middle-income countries. Int J Epidemiol 2013;42:1340–55. 10.1093/ije/dyt109 23920141 PMC3816349

[11] Dewey KG, Begum K Long-term consequences of stunting in early life. Matern Child Nutr 2011;7:5–18. 10.1111/j.1740-8709.2011.00349.x 21929633 PMC6860846

[12] Lelijveld N, Seal A, Wells JC, et al Chronic disease outcomes after severe acute malnutrition in Malawian children (ChroSAM): a cohort study. Lancet Glob Health 2016;4:e654–2. 10.1016/S2214-109X(16)30133-4 27470174 PMC4985564

[13] Mendez MA, Adair LS Severity and timing of stunting in the first two years of life affect performance on cognitive tests in late childhood. J Nutr 1999;129:1555–62. 10.1093/jn/129.8.1555 10419990

[14] Black RE, Allen LH, Bhutta ZA, et al Maternal and child undernutrition: global and regional exposures and health consequences. Lancet 2008;371:243–60. 10.1016/S0140-6736(07)61690-0 18207566

[15] Munthali T, Jacobs C, Sitali L, et al Mortality and morbidity patterns in under-five children with severe acute malnutrition (SAM) in Zambia: a five-year retrospective review of hospital-based records (2009-2013). Arch Public Health 2015;73:23 10.1186/s13690-015-0072-1 25937927 PMC4416273

[16] Burrell A, Kerac M, Nabwera H Monitoring and discharging children being treated for severe acute malnutrition using mid-upper arm circumference: secondary data analysis from rural Gambia. Int Health 2017;9:226–33. 10.1093/inthealth/ihx022 28810666 PMC5881269

[17] Bhutta ZA, Ahmed T, Black RE, et al What works? Interventions for maternal and child undernutrition and survival. Lancet 2008;371:417–40. 10.1016/S0140-6736(07)61693-6 18206226

[18] World Health Organization (WHO). Guideline: updates on the management of severe acute malnutrition in infants and children. Geneva: World Health Organization, 2013. 24649519

[19] Ashraf H, Alam NH, Chisti MJ, et al A follow-up experience of 6 months after treatment of children with severe acute malnutrition in Dhaka, Bangladesh. J Trop Pediatr 2012;58:253–7. 10.1093/tropej/fmr083 21990106

[20] Khanum S, Ashworth A, Huttly SR Growth, morbidity, and mortality of children in Dhaka after treatment for severe malnutrition: a prospective study. Am J Clin Nutr 1998;67:940–5. 10.1093/ajcn/67.5.940 9583853

[21] Kerac M, Bunn J, Chagaluka G, et al Follow-up of post-discharge growth and mortality after treatment for severe acute malnutrition (FuSAM study): a prospective cohort study. PLoS One 2014;9:e96030 10.1371/journal.pone.0096030 24892281 PMC4043484

[22] Isanaka S, Langendorf C, Berthé F, et al Routine Amoxicillin for Uncomplicated Severe Acute Malnutrition in Children. N Engl J Med 2016;374:444–53. 10.1056/NEJMoa1507024 26840134

[23] Berkley JA, Ngari M, Thitiri J, et al Daily co-trimoxazole prophylaxis to prevent mortality in children with complicated severe acute malnutrition: a multicentre, double-blind, randomised placebo-controlled trial. Lancet Glob Health 2016;4:e464–73. 10.1016/S2214-109X(16)30096-1 27265353 PMC6132285

[24] Walker SP, Golden MH Growth in length of children recovering from severe malnutrition. Eur J Clin Nutr 1988;42:395–404. 3135181

[25] Barnett AG, van der Pols JC, Dobson AJ Regression to the mean: what it is and how to deal with it. Int J Epidemiol 2005;34:215–20. 10.1093/ije/dyh299 15333621

[26] Royston P, Sauerbrei W Stability of multivariable fractional polynomial models with selection of variables and transformations: a bootstrap investigation. Stat Med 2003;22:639–59. 10.1002/sim.1310 12590419

[27] Ashraf H, Alam NH, Chisti MJ, et al Observational follow-up study following two cohorts of children with severe pneumonia after discharge from day care clinic/hospital in Dhaka, Bangladesh. BMJ Open 2012;2:e000961 10.1136/bmjopen-2012-000961 PMC4400608 22842561

[28] Costello AM Growth velocity and stunting in rural Nepal. Arch Dis Child 1989;64:1478–82. 10.1136/adc.64.10.1478 2817933 PMC1792803

[29] Heikens GT, Schofield WN, Dawson SM, et al Long-stay versus short-stay hospital treatment of children suffering from severe protein-energy malnutrition. Eur J Clin Nutr 1994;48:873–82. 7889896

[30] Heikens GT, Schofield WN, Dawson S, et al The Kingston project. I. Growth of malnourished children during rehabilitation in the community, given a high energy supplement. Eur J Clin Nutr 1989;43:145–60. 2659312

[31] Richard SA, Black RE, Gilman RH, et al Diarrhea in early childhood: short-term association with weight and long-term association with length. Am J Epidemiol 2013;178:1129–38. 10.1093/aje/kwt094 23966558 PMC3783094

[32] MAL-ED Network Investigators. Relationship between growth and illness, enteropathogens and dietary intakes in the first 2 years of life: findings from the MAL-ED birth cohort study. BMJ Glob Health 2017;2:e000370 10.1136/bmjgh-2017-000370 PMC5759708 29333282

[33] Lima AAM, Soares AM, Filho JQS, et al Enteroaggregative Escherichia coli subclinical infection and coinfections and impaired child growth in the MAL-ED cohort study. J Pediatr Gastroenterol Nutr 2018;66:325–33. 10.1097/MPG.0000000000001717 29356769

[34] Dangour AD, Watson L, Cumming O, et al Interventions to improve water quality and supply, sanitation and hygiene practices, and their effects on the nutritional status of children. Cochrane Database Syst Rev 2013:CD009382 10.1002/14651858.CD009382.pub2 23904195 PMC11608819

[35] Attia S, Versloot CJ, Voskuijl W, et al Mortality in children with complicated severe acute malnutrition is related to intestinal and systemic inflammation: an observational cohort study. Am J Clin Nutr 2016;104:1441–9. 10.3945/ajcn.116.130518 27655441 PMC5081715

[36] Owino V, Ahmed T, Freemark M, et al Environmental Enteric dysfunction and growth failure/stunting in global child health. Pediatrics 2016;138:e20160641 10.1542/peds.2016-0641 27940670

[37] Heikens GT, Schofield WN, Dawson S The Kingston Project. II. The effects of high energy supplement and metronidazole on malnourished children rehabilitated in the community: anthropometry. Eur J Clin Nutr 1993;47:160–73. 8458314

[38] Victora CG, de Onis M, Hallal PC, et al Worldwide timing of growth faltering: revisiting implications for interventions. Pediatrics 2010;125:e473–80. 10.1542/peds.2009-1519 20156903

[39] Dewey KG, Adu-Afarwuah S Systematic review of the efficacy and effectiveness of complementary feeding interventions in developing countries. Matern Child Nutr 2008;4:24–85. 10.1111/j.1740-8709.2007.00124.x 18289157 PMC6860813

[40] Dewey KG The challenge of meeting nutrient needs of infants and young children during the period of complementary feeding: an evolutionary perspective. J Nutr 2013;143:2050–4. 10.3945/jn.113.182527 24132575 PMC3827643

[41] Gupta P, Mital R, Kumar B, et al Physical Growth, morbidity profile and mortality among healthy late preterm neonates. Indian Pediatr 2017;54:629–34. 10.1007/s13312-017-1123-1 28607209

[42] Bhutta ZA, Das JK, Rizvi A, et al Evidence-based interventions for improvement of maternal and child nutrition: what can be done and at what cost? Lancet 2013;382:452–77. 10.1016/S0140-6736(13)60996-4 23746776

[43] Iannotti LL, Lutter CK, Stewart CP, et al Eggs in early complementary feeding and child growth: a randomized controlled trial. Pediatrics 2017;140:e20163459 10.1542/peds.2016-3459 28588101

