# Supplementary material for: Linear growth following complicated severe malnutrition: 1-year follow-up cohort of Kenyan children
Source: Arch Dis Child. 2018 Sep 28;104(3):229–35. doi: 10.1136/archdischild-2018-315641 (PMC6556974; doi:10.1136/archdischild-2018-315641)
Supplement: Supplementary data [file archdischild-2018-315641supp001.pdf]

Primary Author & reference	Year	Location	Intervention	Complicated	Age months	N	Intervention group 1 HAZ mean	Intervention group 2 HAZ mean	Reference group HAZ mean	Follow up duration	Intervention group 1 HAZ mean change	Intervention group 2 HAZ mean change	Reference group HAZ mean change
Bahwere *et. al. (a)*[1]	2017	Malawi	Milk free RUTF	No	6-23	795	-3.1	-3.0	-3.3	90 days	Not provided	Not provided	Not provided
Bahwere *et. al. (b)*[1]	2017	Malawi	Milk free RUTF	No	24-59	504	-3.6	-3.1	-3.6	90 days	Not provided	Not provided	Not provided
Versloot *et. al.*[2]	2017	Malawi	RUTF + F75	Yes	6-60	74	-2.8	-3.2	-3.6	Inpatient treatment	Not provided	Not provided	Not provided
Grellety et.al.[3]	2017	Congo DR	Cash transfers$	No	6-59	1481	-3.4	N/A	-3.5	6 months	-0.15	N/A	-0.18
Isanaka *et. al.*[4]	2016	Niger	Antibiotics	No	6-59	2399	-3.0	N/A	-3.0	8 weeks	Not provided	N/A	Not provided
Bahwere *et. al. (a)*[5]	2016	Congo DR	Milk free RUTF	No	6-23	407	-4.3	N/A	-4.0	Outpatient treatment	-0.12	N/A	-0.15
Bahwere *et. al. (b)*[5]	2016	Congo DR	Milk free RUTF	No	24-59	468	-4.8	N/A	-5.0	Outpatient treatment	0.05	N/A	0.01
Berkley et. al.[6]	2016	Kenya	Antibiotics	Yes	2-59	1778	-2.8	N/A	-2.9	12 months	-0.02	N/A	0.00
Binns et.al.[7]	2016	Malawi	Discharge	No	6-51	258	Not provided	N/A	Not provided	N/A	Not provided	N/A	Not provided
Denoeud-Ndam et.al.[8]	2016	Mali & Niger	Antimalarial	No	6-59	133	Not provided	N/A	Not provided	42 days	Not provided	N/A	Not provided
Bhandari et.al.[9]	2016	India	Local RUTF & foods	No	6-59	906	-2.9	-3.1	-3.0	16 weeks	0.04	0.07	0.08
Hseih et.al.[10]	2015	Malawi	Fatty acids	No	6-59	141	-3.3	N/A	-2.9	4 weeks	Not provided	N/A	Not provided
Jones et.al.[11]	2015	Kenya	Essential fatty acids	No	6-50	60	-3.4	-2.6	-3.1	84 days	Not provided	N/A	Not provided
Bahwere *et. al.*[12]	2014	Malawi	Essential fatty acids	No	6-59	595	-3.3	N/A	-3.3	84 days	Not provided	N/A	Not provided
Trehan et.al.[13]	2013	Malawi	Antibiotics	No	6-59	2767	-3.1	-3.2	-3.2	3 months	Not provided	N/A	Not provided
Nahar et.al.[14]	2012	Bangladesh	Psychosocial stimulation	No	6-24	507	-3.4	-3.6	-3.6	6 months	Not provided	N/A	Not provided
Akech et,al.[15]	2010	Kenya	Fluids/shock	Yes	>6	61	Not provided	N/A	Not provided	Inpatient treatment	Not provided	N/A	Not provided
Kerac et.al.[16]	2009	Malawi	Probiotics	Yes	5-168	795	-3.2	N/A	-3.1	Inpatient & outpatient	Not provided*	N/A	Not provided*
Dubray et.al.[17]	2008	Sudan	Antibiotics	No	6-59	458	Not provided	N/A	Not provided	14 days	Not provided	N/A	Not provided

a,b; trial where analysis were categorised into two groups (6-23 and 24-59 months), $; cluster-randomized trial, N/A-trials with only two arms
* the study population was also followed up ~1 year after the end of nutritional rehabilitation, pooled HAZ -2.97 (https://doi.org/10.1371/journal.pone.0096030)

Supplementary Table 1: **Listing of clinical trials in severe acute malnutrition showing height/length-for-age z-scores.**

	**Study enrolment**	**Month 1**	**Month 2**	**Month 3**	**Month 4**	**Month 5**	**Month 6**	**Month 8**	**Month 10**	**Month 12**
**Changes in HAZ categories**										
**HAZ Groups**	N=1169	N=1130	N=1145	N=1103	N=1091	N=1077	N=1129	N=1065	N=1045	N=1169
HAZ≥-2	358 (31)	287 (25)	281 (25)	276 (25)	272 (25)	274 (25)	298 (26)	262 (25)	250 (24)	296 (25)
-2 to -3	290 (25)	276 (24)	285 (25)	292 (26)	298 (27)	309 (29)	320 (28)	302 (28)	319 (31)	358 (31)
HAZ <-3	521 (44)	567 (50)	579 (51)	535 (49)	521 (48)	494 (46)	511 (45)	501 (47)	476 (46)	515 (44)
**Changes in WHZ categories**										
**WHZ Groups**	N=959*	N=1118	N=1138	N=1102	N=1086	N=1072	N=1126	N=1063	N=1042	N=1167
WHZ≥-2	119 (12)	429 (38)	595 (52)	636 (58)	664 (61)	653 (61)	742 (66)	746 (70)	747 (72)	869 (74)
-2 to -3	246 (26)	312 (28)	281 (25)	241 (22)	242 (22)	234 (22)	218 (19)	178 (17)	183 (18)	182 (16)
WHZ<-3	594 (62)	377 (34)	262 (23)	225 (20)	180 (17)	185 (17)	166 (15)	139 (13)	112 (11)	116 (9.9)
**Changes in WAZ categories**										
**WAZ Groups**	N=959*	N=1121	N=1138	N=1104	N=1090	N=1075	N=1128	N=1063	N=1043	N=1167
WAZ≥-2	24 (2.5)	128 (11)	205 (18)	251 (23)	272 (25)	312 (29)	354 (31)	385 (36)	394 (38)	486 (42)
-2 to -3	143 (15)	249 (22)	312 (27)	322 (29)	353 (32)	337 (31)	365 (32)	318 (30)	321 (31)	343 (29)
WAZ<-3	792 (83)	744 (66)	621 (55)	531 (48)	465 (43)	426 (40)	409 (36)	360 (34)	328 (31)	338 (29)
**Changes in MUAC (cm) categories**										
MUAC Groups	N=1169	N=1151	N=1150	N=1133	N=1118	N=1111	N=1143	N=1098	N=1079	N=1169
≥12.5cm	37 (3.2)	156 (14)	337 (29)	436 (38)	532 (48)	568 (51)	655 (57)	718 (65)	778 (72)	906 (78)
11.5 to 12.5cm	72 (6.2)	330 (29)	389 (34)	374 (33)	339 (30)	336 (30)	315 (28)	246 (22)	207 (19)	189 (16)
<11.5cm	1060 (91)	665 (58)	424 (37)	323 (29)	247 (22)	207 (19)	173 (15)	134 (12)	94 (8.7)	74 (6.3)

*the N<1169 because 210 children had edema (there are no WHZ & WAZ for children with edema), HAZ-height-for-age z-score, WAZ-weight-for-age z-score, WHZ-weight-for-height/length z-score, MUAC-mid-upper arm circumference, there were no scheduled follow-ups at months 7, 9 and 11

Supplementary Table 2: **Monthly changes in HAZ, WHZ, WAZ and MUAC categories.**

	HAZ at enrolment			
**Demographic data at study enrolment**	**HAZ>-2 (N=358)**	**HAZ -3 to -2 (N=290)**	**HAZ <-3 (N=521)**	***P*-value**
Sex (female)	215 (60)	148 (51)	202 (39)	<0.001
Age in months, median (IQR)	9 (7-13)	11 (8-17)	13 (7-21)	0.0001
Main caregiver-not biological parent	19 (5.3)	17 (5.9)	45 (8.6)	0.12
Randomized to co-trimoxazole prophylaxis	180 (50)	152 (52)	265 (51)	0.86
Born underweight[1]	34 (9.5)	44 (15)	151 (29)	<0.001
Born premature[2]	21 (5.9)	30 (10)	90 (17)	<0.001
**Index admission diagnosis**				
Index admission with clinical signs of rickets	33 (9.2)	34 (12)	72 (14)	0.12
Index admission with diarrhoea	229 (64)	170 (59)	280 (54)	0.01
Index admission with severe pneumonia	140 (39)	99 (34)	165 (32)	0.07
Index admission with tuberculosis	11 (3.1)	17 (5.9)	17 (3.3)	0.12
Other co-mobidities[3]	11 (3.1)	16 (5.5)	39 (7.5)	0.02
**Nutritional status**				
Oedema at study enrolment	38 (11)	47 (16)	125 (24)	<0.001
MUAC (cm) at study enrolment, mean ±*SD*	10.9 ±0.8	10.8 ±0.9	10.4 ±1.2	<0.001
Height difference between enrolment and month 1, mean ±*SD*	0.39 ±1.2	0.57 ±1.3	0.83 ±1.3	<0.001
Height difference between enrolment and month 12, mean ±*SD*	10.63 ±3.4	10.28 ±3.5	11.0 ±4.2	0.03
HAZ difference between enrolment and month 1, mean ±*SD*	-0.38 ±0.5	-0.22 ±0.5	-0.08 ±0.5	<0.001
HAZ difference between enrolment and month 12, mean ±*SD*	-0.63 ±0.9	-0.16 ±0.8	0.51 ±1.0	<0.001
**Follow-up illness events**				
Outpatient treatment for diarrhoea	114 (32)	78 (27)	137 (26)	0.17
Outpatient treatment for pneumonia	97 (27)	72 (25)	106 (20)	0.06
Outpatient treatment for other diagnosis[4]	129 (36)	87 (30)	171 (33)	0.26
Re-admission for diarrhoea	31 (8.7)	35 (12)	36 (6.9)	0.04
Re-admission for severe pneumonia	43 (12)	40 (14)	76 (15)	0.55
Re-admission for other diagnosis[4]	33 (9.2)	24 (8.3)	39 (7.5)	0.66

HAZ-height-for-age z-score, MUAC- mid-upper arm circumference, [1]birth weight <2500g, [2]gestational age <37 weeks, [3]these were chronic illness at study enrolment including 5 sickle cell, 15 heart diseases, 38 cerebral palsy, 3 epilepsy and 5 children with both cerebral palsy and epilepsy, [4]these were diagnosis of Malaria, tuberculosis, sepsis, meningitis, measles, anaemia and urinary tract infection and skin/soft tissue infection, the ages, MUAC and HAZ difference were compared using Kruskal-Wallis test, the other p-values are from chi-square test, *SD*-standard deviation.

Supplementary Table 3: **Distribution of the enrolment and follow-up illness features stratified by baseline HAZ groups.**

**Demographic variables**	**Adjusted Odds Ratio**	***95% CI***	***P*-value**
Sex (female)	0.48	0.36-0.63	<0.001
Age in months			
≥24		Reference	
12 to 23	0.20	0.09-0.40	<0.001
6 to 23	0.09	0.04-0.18	<0.001
<6	0.07	0.03-0.17	<0.001
Recruitment Hospital			
Kilifi County Hospital		Reference	
Coast General Hospital	0.31	0.17-0.56	<0.001
Malindi sub-county hospital	0.90	0.45-1.83	0.78
Mbagathi sub-county hospital	0.62	0.33-1.18	0.15
Born underweight[1]	3.27	2.15-4.98	<0.001
**Nutritional status**			
Oedema at study enrolment	2.21	1.33-3.67	0.002
MUAC (cm) at study enrolment	0.47	0.39-0.57	<0.001

HAZ-height-for-age z-score, MUAC- mid-upper arm circumference, [1]birth weight <2500g, the multivariable logistic regression compared children stunted (HAZ<-2) against non-stunted (HAZ≥-2) at baseline, *P*-values from stepwise multivariable logistic regression.

Supplementary Table 4: **Factors at study enrolment associated with baseline stunting (HAZ <-2Z)**

	Height (cm)		HAZ		Weight (kg)		WAZ		WHZ		MUAC (cm)	
	**Mean ±*SD***	**Change ±*SD****	**Mean ±*SD***	**Change ±*SD****	**Mean ±*SD***	**Change ±*SD****	**Mean ±*SD***	**Change ±*SD****	**Mean ±*SD***	**Change ±*SD****	**Mean ±*SD***	**Change ±*SD****
Enrolment	67.9 ±8.1	-	-2.87 ±1.6	-	5.8 ±1.6	-	-3.96 ±1.1	-	-3.37 ±1.3	-	10.6 ±1.1	-
Month 1	68.6 ±7.7	6.5 ±12.8	-3.06 ±1.5	-0.20 ±0.5	6.4 ±1.7	574 ±6.1	-3.54 ±1.3	0.42 ±0.7	-2.58 ±1.5	0.79 ±1.0	11.4 ±1.3	8.00 ±8.2
Month 2	69.8 ±7.5	11.8 ±11.7	-3.04 ±1.5	0.05 ±0.5	6.9 ±1.7	477±4.9	-3.20 ±1.3	0.34 ±0.6	-2.17 ±1.4	0.42 ±0.9	11.9 ±1.3	5.29 ±6.7
Month 3	70.8 ±7.2	10.3 ±12.5	-3.01 ±1.4	0.03 ±0.5	7.2 ±1.7	329 ±4.0	-3.02 ±1.3	0.17 ±0.4	-1.98 ±1.5	0.17 ±0.7	12.2 ±1.3	2.91 ±5.7
Month 4	72.0 ±6.9	11.9 ±11.3	-2.94 ±1.4	0.08 ±0.4	7.5 ±1.7	303 ±3.7	-2.87 ±1.3	0.15 ±0.4	-1.86 ±1.4	0.12 ±0.7	12.5 ±1.3	2.43 ±5.7
Month 5	73.0 ±6.8	10.1 ±10.0	-2.89 ±1.4	0.04 ±0.4	7.8 ±1.8	250 ±3.8	-2.77±1.3	0.09 ±0.4	-1.79 ±1.4	0.06 ±0.6	12.7 ±1.3	1.77 ±5.3
Month 6	74.0 ±6.7	10.2 ±10.5	-2.86 ±1.4	0.05 ± 0.4	8.1 ±1.8	236 ±3.7	-2.70 ±1.3	0.07 ±0.4	-1.77 ±1.4	0.04 ±0.6	12.8 ±1.3	1.63 ±5.1
Month 8	75.6 ±6.7	16.1 ±13.5	-2.91 ±1.4	-0.02 ±0.5	8.5 ±1.8	416 ±4.8	-2.60 ±1.3	0.10 ±0.5	-1.59 ±1.4	0.17 ±0.7	13.1 ±1.4	2.57 ±6.3
Month 10	77.2 ±6.7	15.4 ±12.6	-2.92 ±1.4	-0.01 ±0.4	8.9 ±1.9	386 ±4.6	-2.54 ±1.3	0.06 ±0.4	-1.49 ±1.4	0.11 ±0.6	13.3 ±1.4	2.56 ±5.5
Month 12	78.8 ±6.4	16.4 ±11.8	-2.88 ±1.4	0.05 ±0.4	9.3 ±1.9	378 ±5.0	-2.47 ±1.3	0.08 ±0.4	-1.39 ±1.2	0.10± 0.7	13.5 ±1.4	1.94 ±5.7

Results are means and standard deviation, HAZ-height-for-age z-score, WAZ-weight-for-age z-score, WHZ-weight-for-height/length z-score, MUAC-mid-upper arm circumference

Supplementary Table 5: **Monthly changes in anthropometry during the one-year follow-up.**

	Change in HAZ between enrolment and month 12		
**Demographic data at study enrolment**	**HAZ change -0.25 to 0.25 (N=262)**	**HAZ loss >0.25 (N=472)**	**HAZ gain >0.25 (N=435)**
Sex (female)	130 (50)	244 (52)	191 (44)
Age in months, median (IQR)	13 (8-18)	10 (7-14)	12 (7-20)
24 to 59 months	36 (14)	29 (6.1)	74 (17)
12 to 23 months	110 (42)	154 (33)	151 (35)
6 to 11 months	86 (33)	224 (47)	125 (29(
2 to 5 months	30 (11)	65 (14)	85 (20)
Recruitment hospital			
Kilifi County Hospital	27 (10)	40 (8.5)	47 (11)
Coast General Hospital	122 (47)	255 (54)	165 (38)
Malindi sub-county hospital	48 (18)	66 (14)	88 (20)
Mbagathi sub-county hospital	65 (25)	111 (24)	135 (31)
Main caregiver-not biological parent	11 (4.2)	29 (6.1)	41 (9.5)
Randomized to co-trimoxazole prophylaxis	150 (57)	236 (50)	211 (49)
Born underweight[1]	44 (17)	71 (15)	114 (26)
Born premature[2]	24 (9.2)	37 (7.8)	80 (18)
**Index admission diagnosis**			
Index admission with clinical signs of rickets	26 (9.9)	43 (9.1)	70 (16)
Index admission with diarrhoea	153 (58)	283 (60)	243 (56)
Index admission with severe pneumonia	79 (30)	175 (37)	150 (34)
Index admission with tuberculosis	9 (3.4)	14 (3.0)	22 (5.1)
Other co-mobidities[3]	17 (6.5)	28 (5.9)	21 (4.8)
**Nutritional status at study enrolment**			
Oedema	52 (20)	65 (14)	93 (21)
Height/length-for-age z score, mean ±*SD*	-3.0 ±1.3	-2.0 ±1.5	-3.7 ±1.4
Mid-upper arm circumference (cm), mean ±*SD*	10.7 ±1.0	10.8 ±0.9	10.4 ±1.2
**Follow-up illness events**			
Outpatient treatment for diarrhoea	62 (24)	150 (32)	117 (27)
Outpatient treatment for pneumonia	62 (24)	114 (24)	99 (23)
Outpatient treatment for other diagnosis[4]	92 (24)	154 (40)	141 (36)
Re-admission for diarrhoea	16 (6.1)	55 (12)	31 (7.1)
Re-admission for severe pneumonia	27 (10)	78 (17)	54 (12)
Re-admission for other diagnosis[4]	21 (22)	42 (44)	33 (34)

HAZ-height-for-age z-score, HAZ diff-the difference between HAZ at study conclusion and at study enrolment, RRR-relative risk ratios, [1]birth weight <2500g, [2]gestation age <37 weeks, [3]these were chronic illness at study enrolment including 5 sickle cell, 15 heart diseases, 38 cerebral palsy, 3 epilepsy and 5 children with both cerebral palsy and epilepsy, , [4]these were diagnosis of Malaria, tuberculosis, sepsis, meningitis, measles, anemia and urinary tract infection and skin/soft tissue infection, Relative risk ratios are computed using multinomial logistic regression with HAZ diff -0.25 to 0.25 as the reference.

Supplementary Table 6: **Distribution of characteristics at enrolment and follow-up illness events, stratified by the HAZ change groups.**

	Lost at least 0.25 HAZ *			Gained at least 0.25 HAZ *		
**Demographics at study enrolment**	**Crude RRR**	**95% CI**	**P-value**	**Crude RRR**	**95% CI**	**P-value**
Sex (female)	1.09	0.80-1.47	0.59	0.79	0.58-1.08	0.14
Age in Months						
Age 24 to 59 months	1.0	Reference		1.0	Reference	
Age 12 to 23 months	1.74	1.01-3.01	0.04	0.67	0.42-1.07	0.09
Age 6 to 11 months	3.23	1.87-5.60	<0.001	0.71	0.44-1.15	0.16
Age 2 to 5 months	2.69	1.40-5.17	0.003	1.38	0.77-2.45	0.28
Recruitment Hospital						
Kilifi County Hospital	1.0	Reference		1.0	Reference	
Coast General Hospital	1.41	0.83-2.41	0.21	0.78	0.46-1.32	0.35
Malindi sub-county hospital	0.93	0.50-1.71	0.81	1.05	0.58-1.90	0.86
Mbagathi sub-county hospital	1.15	0.65-2.05	0.63	1.19	0.68-2.08	0.54
Main caregiver-not mother	1.49	0.73-3.04	0.27	2.37	1.20-4.71	0.01
Randomized to co-trimoxazole prophylaxis	0.75	0.55-1.01	0.06	0.70	0.52-0.96	0.03
Born underweight[1]	0.86	0.57-1.30	0.47	1.78	1.20-2.63	0.004
Born premature[2]	0.85	0.50-1.46	0.56	2.27	1.40-3.69	0.001
**Index admission diagnosis**						
Index admission with clinical signs of rickets	0.91	0.55-1.52	0.72	1.74	1.08-2.81	0.02
Index admission with diarrhoea	1.07	0.78-1.45	0.68	0.90	0.66-1.23	0.51
Index admission with severe pneumonia	1.36	0.97-1.89	0.06	1.22	0.88-1.69	0.24
Index admission with tuberculosis	0.86	0.37-2.01	0.73	1.50	0.68-3.30	0.32
Other co-mobidities[3]	0.91	0.49-1.96	0.76	0.73	0.38-1.41	0.35
**Nutritional status at study enrolment**						
Oedema	0.64	0.43-0.97	0.04	1.10	0.75-1.60	0.63
Height/length-for-age z score per unit z-score	1.66	1.48-1.87	<0.001	0.70	0.63-0.78	<0.001
Mid-upper arm circumference per cm	1.03	0.89-1.19	0.70	0.73	0.62-0.85	<0.001
**Follow-up illness events**						
Outpatient treatment for diarrhoea	1.50	1.06-2.12	0.02	1.19	0.83-1.69	0.34
Outpatient treatment for pneumonia	1.03	0.72-1.46	0.88	0.95	0.66-1.37	0.78
Outpatient treatment for other diagnosis[4]	0.89	0.65-1.23	0.49	0.89	0.65-1.22	0.46
Re-admission for diarrhoea	2.03	1.14-3.62	0.02	1.18	0.63-2.20	0.60
Re-admission for severe pneumonia	1.72	1.08-2.75	0.02	1.23	0.76-2.01	0.40
Re-admission for other diagnosis[4]	1.12	0.65-1.94	0.68	0.94	0.53-1.67	0.84

* compared to children with minimal change in HAZ (+/-<0.25Z). HAZ-height-for-age z-score, HAZ diff-the difference between HAZ at study conclusion and at study enrolment, RRR-relative risk ratios, [1]birth weight <2500g, [2]gestation age <37 weeks, [3]these were chronic illness at study enrolment including 5 sickle cell, 15 heart diseases, 38 cerebral palsy, 3 epilepsy and 5 children with both cerebral palsy and epilepsy, [4]these were diagnosis of malaria, tuberculosis, sepsis, meningitis, measles, anaemia and urinary tract infection and skin/soft tissue infection, Relative risk ratios are computed using multinomial logistic regression with HAZ diff -0.25 to 0.25 as the reference, P-values from crude multinomial logistic regression models.

Supplementary Table 7: **Univariate analysis of factors associated with loss or gain of HAZ during the one-year follow-up.**

	Lost at least 0.25 HAZ[a]			Gained at least 0.25 HAZ[a]		
**Demographics at study enrolment**	**Adjusted RRR**	**95% CI**	**P-value**	**Adjusted RRR**	**95% CI**	**P-value**
Age in months						
≥24		Reference			Reference	
12-23	1.16	0.61-2.19	0.66	0.56	0.34-0.95	0.03
6-23	2.05	1.04-4.02	0.03	0.63	0.35-1.11	0.11
<6	1.97	0.81-4.79	0.14	1.00	0.47-2.12	0.99
Main caregiver not the biological mother	-	-	-	2.81	1.26-6.26	0.01
Randomised to co-trimoxazole prophylaxis	0.52	0.35-0.77	0.001	0.52	0.37-0.75	<0.001
Born prematurely[b]	-	-	-	2.10	1.24-3.58	0.006
MUAC (cm)	-	-	-	0.79	0.66-0.95	0.01
**Follow-up illness events**						
Outpatient treatment for diarrhoea	1.60	1.01-2.53	0.04	-	-	-
Re-admission to hospital for diarrhoea	2.13	1.04-4.36	0.03	-	-	-
Re-admission to hospital for severe pneumonia	1.88	1.03-3.45	0.04	-	-	-

[a]compared to children with minimal change in HAZ (± 0.25Z). HAZ-height-for-age z-score, HAZ difference-the difference between HAZ at month 12 and at study enrolment, RRR-relative risk ratios, [b]gestational age <37 weeks, Relative risk ratios are computed using multinomial logistic regression with HAZ difference -0.25 to 0.25 as the reference and adjusted for regression to the mean, P-values from multivariable multinomial logistic regression, only children who were stunted (HAZ<-2) at baseline were included in the analysis.

Supplementary Table 8: **Factors associated with loss or gain of HAZ during the one-year follow-up amongst children who were stunted at baseline.**

	WHZ <-1.3 at month 1 (N=894)	WHZ ≥-1.3 at month 1 (N=275)	*P*-value
**Demographics at study enrolment**			
Sex (female)	433 (48)	132 (48)	0.90
Age in months-median (*IQR*)	11 (7-16)	12 (6-21)	0.20
Main caregiver-not mother	59 (6.6)	22 (8.0)	0.42
Randomized to co-trimoxazole prophylaxis	458 (51)	139 (51)	0.84
Born prematurely[1]	102 (11)	39 (14)	0.41
**Index admission diagnosis**			
Index admission with clinical signs of rickets	119 (13)	20 (7.3)	0.007
Index admission with diarrhoea	522 (58)	157 (57)	0.70
Index admission with severe pneumonia	331 (37)	73 (27)	0.001
Index admission with tuberculosis	41 (4.6)	4 (1.5)	0.01
Other co-mobidities[2]	58 (6.5)	8 (2.9)	0.03
**Nutritional status at study enrolment**			
Oedema	120 (13)	90 (33)	<0.001
Height/length-for-age z score, mean ± *SD*	-2.77 ± 1.6	-3.18 ± 1.6	0.0003
Weight-for-height z score, mean ± *SD*	-3.58 ± 1.1	-2.23 ± 1.3	<0.001
Weight-for-age z-score, mean ± *SD*	-4.02 ± 1.0	-3.69 ± 1.1	0.0002
Mid-upper arm circumference (cm), mean ± *SD*	10.51 ± 0.9	11.01 ± 1.3	<0.001
**Changes in height/length z-scores (HAZ)**			
Change in HAZ between enrolment and month 1	-0.20 ± 0.5	-0.22 ± 0.6	0.40
Change in HAZ between month 1 and month 3	-0.02 ± 0.5	0.38 ± 0.7	<0.001
Change in HAZ between month 3 and month 12	0.13 ± 0.8	0.21 ± 0.8	0.23
**Height/length z-scores (HAZ) at month 12**			
Height/length-for-age z score, mean ± *SD*	-2.88 ± 1.4	-2.87 ± 1.3	0.82

WHZ-weight-for-height/length z-score, [1] gestational age <37 weeks, [2]these were chronic illness at study enrolment including 5 sickle cell, 15 heart diseases, 38 cerebral palsy, 3 epilepsy and 5 children with both cerebral palsy and epilepsy, all continuous variables were compared using Wilcoxon rank-sum test and categorical using chi-square test.

Supplementary Table 9: **Distribution of enrolment features and month 12 HAZ stratified by a WHZ threshold of -1.3 at month 1 of follow-up.**

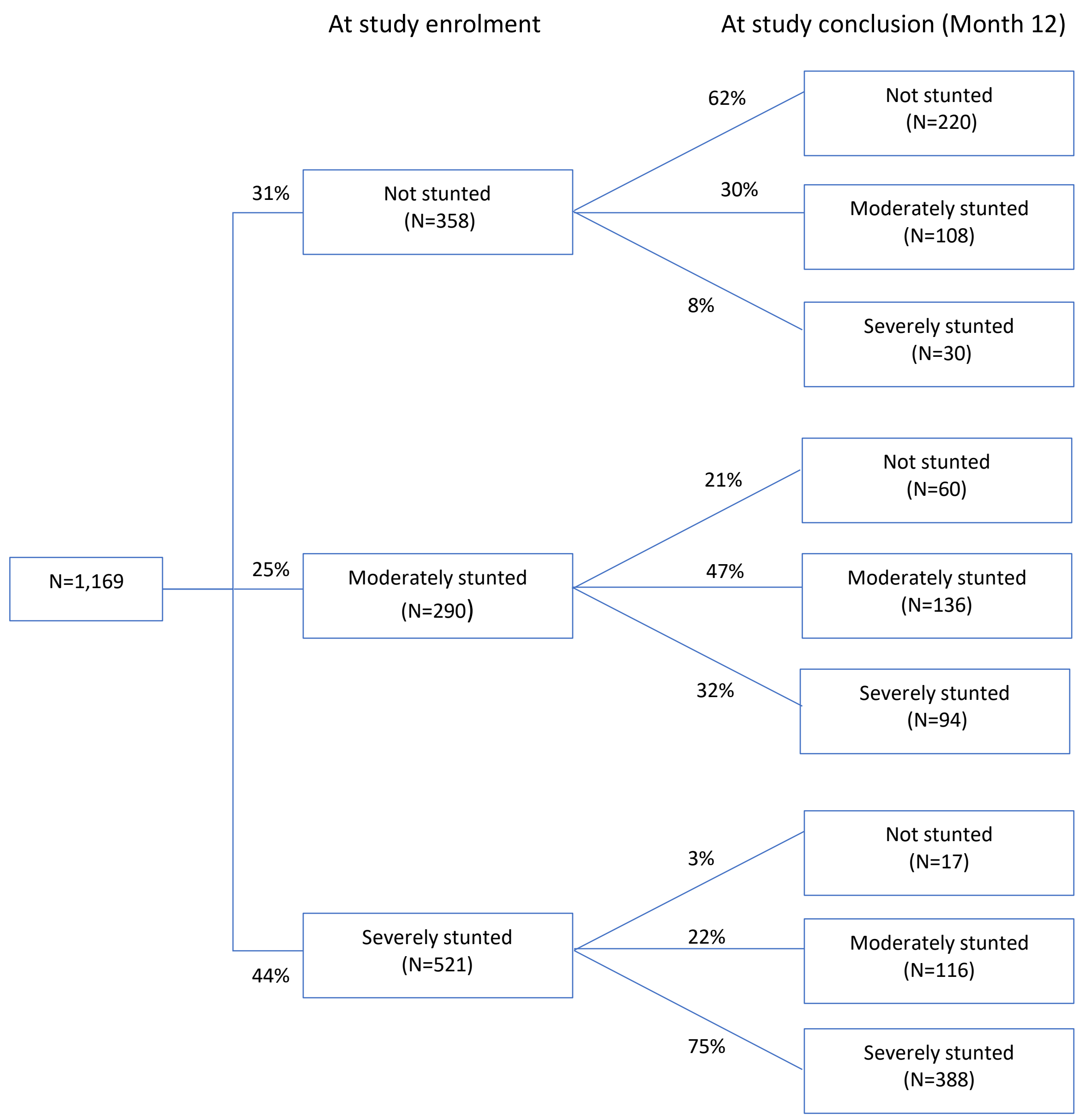

HAZ-height-for-age z-score, the percentages at study enrolment are proportion of children in each group of total children analysed (N=1,169), percentages at study conclusion are proportion of children in each group of total children in the proceeding group.

Supplementary Figure 1: **HAZ categories at study conclusion by HAZ categories at study enrolment.**

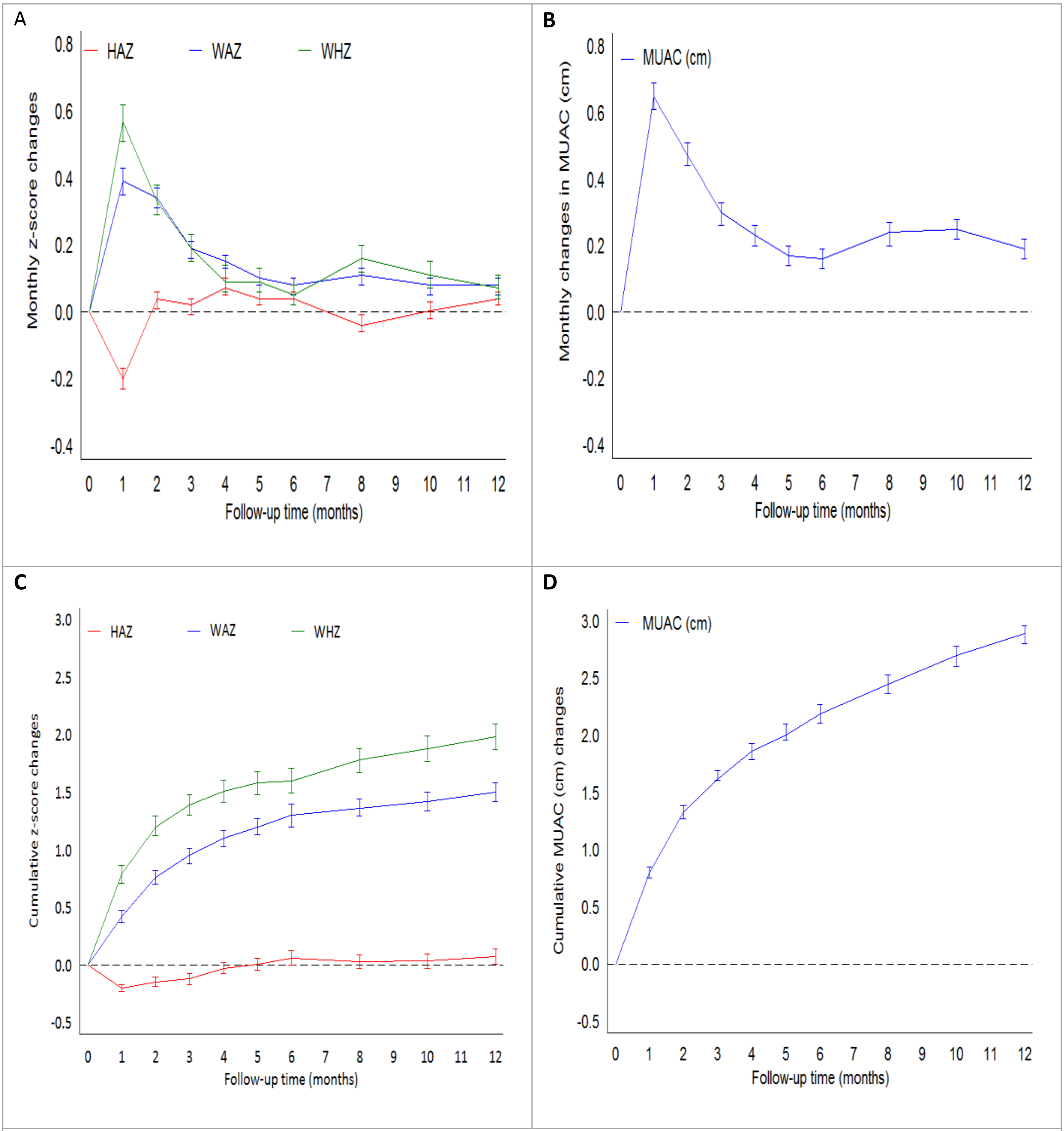

The lines are the mean changes means with 95% confidence interval, the black dashed line represent no change (Y=0). The cumulative change is the difference between monthly values and the study enrolment values.

Supplementary Figure 2: **A-HAZ, WAZ and WHZ monthly z-scores changes, B-Monthly MUAC (cm) changes, C-Cumulative changes in HAZ, WAZ and WHZ, and D-Cumulative changes in MUAC (cm).**

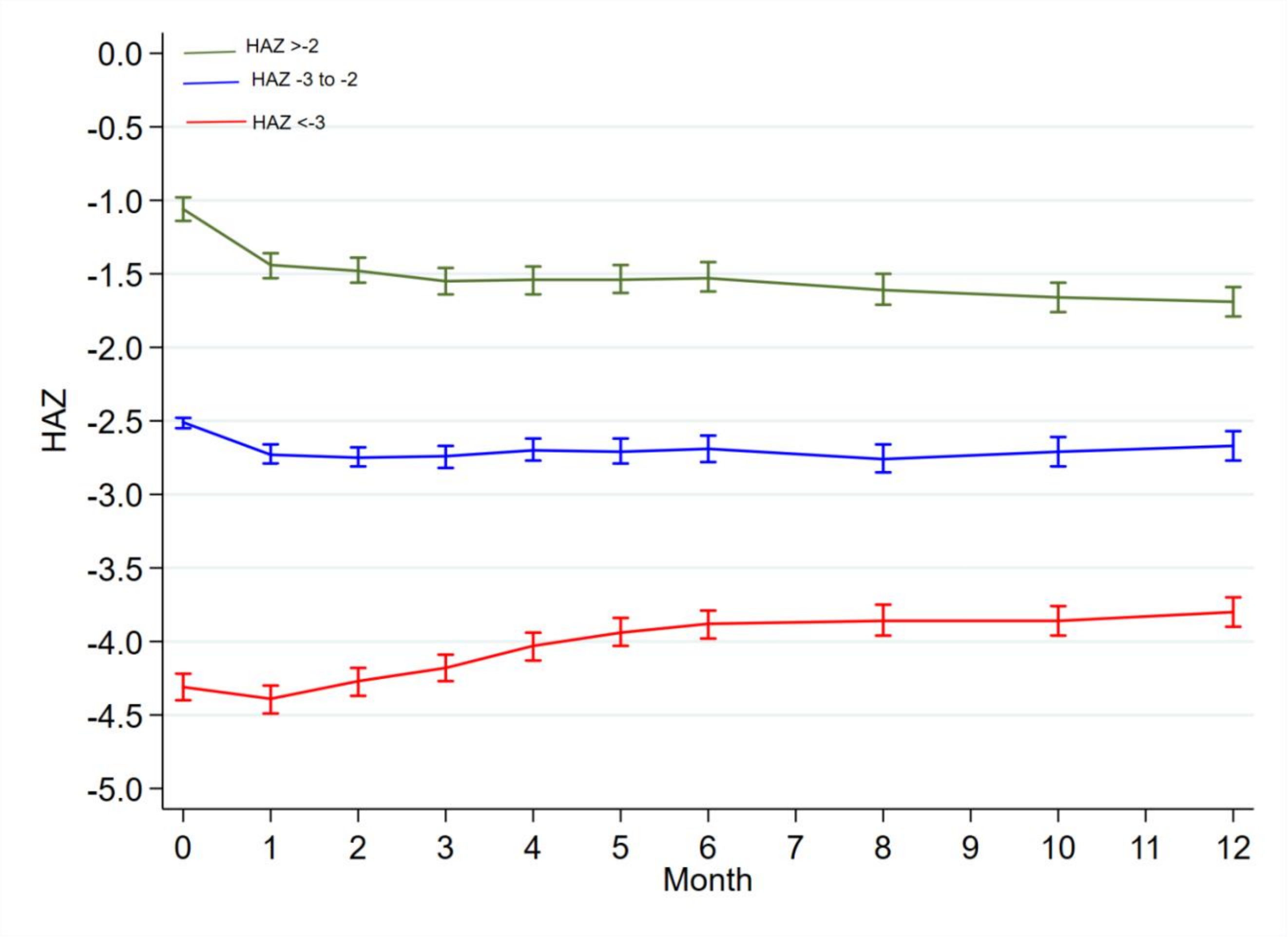

HAZ-height-for-age z-score, there were no anthropometry collected at months 7, 9 and 11, the plotted data are means and *95% CI*.

Supplementary Figure 3: **HAZ trajectory stratified by HAZ group at study enrolment.**

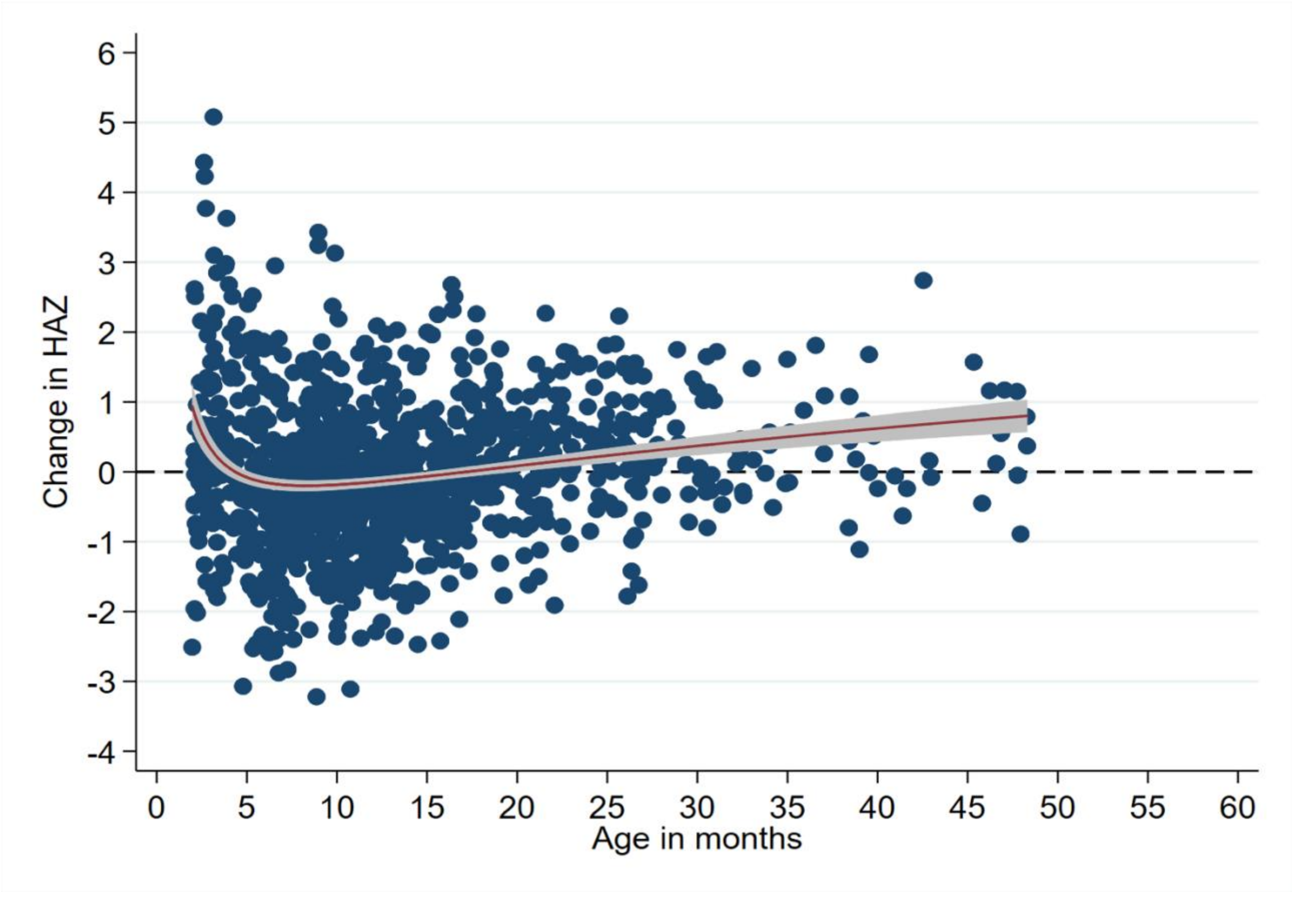

HAZ-height/length-for-age z-score, change in HAZ is the difference between month 12 follow-up and enrolment values. P<0.001 for comparison of model deviance between the fractional polynomial shown and a linear model.

Supplementary Figure 4: **Scatter plot of change in HAZ with age in months with its fitted fractional polynomial curve (*95% CI*).**

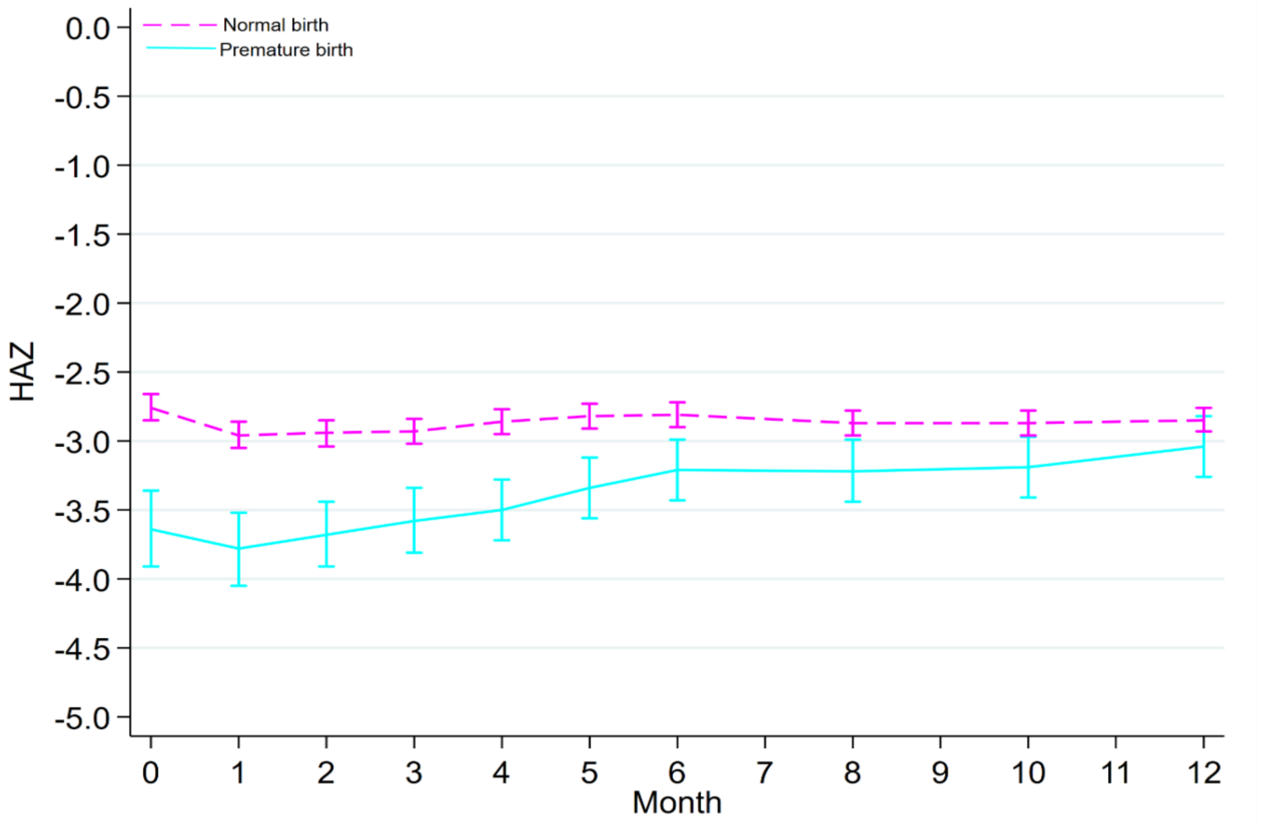

HAZ-height-for-age z-score, Premature-children born at gestational age <37 weeks, there were no anthropometry collected at months 7, 9 and 11, the plotted data are means and *95% CI*.

Supplementary Figure 5: **HAZ trajectory of children born prematurely and those born at term.**

References

1. Bahwere P, Akomo P, Mwale M, Murakami H, Banda C, Kathumba S, et al. Soya, maize, and sorghum-based ready-to-use therapeutic food with amino acid is as efficacious as the standard milk and peanut paste-based formulation for the treatment of severe acute malnutrition in children: a noninferiority individually randomized controlled efficacy clinical trial in Malawi. *The American journal of clinical nutrition*. 2017;106(4):1100-1112.
2. Versloot CJ, Voskuijl W, van Vliet SJ, van den Heuvel M, Carter JC, Phiri A, et al. Effectiveness of three commonly used transition phase diets in the inpatient management of children with severe acute malnutrition: a pilot randomized controlled trial in Malawi. *BMC pediatrics*. 2017;17(1):112.
3. Grellety E, Babakazo P, Bangana A, Mwamba G, Lezama I, Zagre NM, et al. Effects of unconditional cash transfers on the outcome of treatment for severe acute malnutrition (SAM): a cluster-randomised trial in the Democratic Republic of the Congo. *BMC medicine*. 2017;15(1):87.
4. Isanaka S, Langendorf C, Berthe F, Gnegne S, Li N, Ousmane N, et al. Routine Amoxicillin for Uncomplicated Severe Acute Malnutrition in Children. *The New England journal of medicine*. 2016;374(5):444-453.
5. Bahwere P, Balaluka B, Wells JC, Mbiribindi CN, Sadler K, Akomo P, et al. Cereals and pulse-based ready-to-use therapeutic food as an alternative to the standard milk- and peanut paste-based formulation for treating severe acute malnutrition: a noninferiority, individually randomized controlled efficacy clinical trial. *The American journal of clinical nutrition*. 2016;103(4):1145-1161.
6. Berkley JA, Ngari M, Thitiri J, Mwalekwa L, Timbwa M, Hamid F, et al. Daily co-trimoxazole prophylaxis to prevent mortality in children with complicated severe acute malnutrition: a multicentre, double-blind, randomised placebo-controlled trial. *The Lancet Global health*. 2016;4(7):e464-473.
7. Binns PJ, Dale NM, Banda T, Banda C, Shaba B, Myatt M. Safety and practicability of using mid-upper arm circumference as a discharge criterion in community based management of severe acute malnutrition in children aged 6 to 59 months programmes. *Archives of public health = Archives belges de sante publique*. 2016;74:24.
8. Denoeud-Ndam L, Dicko A, Baudin E, Guindo O, Grandesso F, Diawara H, et al. Efficacy of artemether-lumefantrine in relation to drug exposure in children with and without severe acute malnutrition: an open comparative intervention study in Mali and Niger. *BMC medicine*. 2016;14(1):167.
9. Bhandari N, Mohan SB, Bose A, Iyengar *SD*, Taneja S, Mazumder S, et al. Efficacy of three feeding regimens for home-based management of children with uncomplicated severe acute malnutrition: a randomised trial in India. *BMJ Glob Health*. 2016;1(4):e000144.
10. Hsieh JC, Liu L, Zeilani M, Ickes S, Trehan I, Maleta K, et al. High-Oleic Ready-to-Use Therapeutic Food Maintains Docosahexaenoic Acid Status in Severe Malnutrition. *J Pediatr Gastroenterol Nutr*. 2015;61(1):138-143.
11. Jones KD, Ali R, Khasira MA, Odera D, West AL, Koster G, et al. Ready-to-use therapeutic food with elevated n-3 polyunsaturated fatty acid content, with or without fish oil, to treat severe acute malnutrition: a randomized controlled trial. *BMC medicine*. 2015;13:93.
12. Bahwere P, Banda T, Sadler K, Nyirenda G, Owino V, Shaba B, et al. Effectiveness of milk whey protein-based ready-to-use therapeutic food in treatment of severe acute malnutrition in Malawian under-5 children: a randomised, double-blind, controlled non-inferiority clinical trial. *Maternal & child nutrition*. 2014;10(3):436-451.
13. Trehan I, Goldbach HS, LaGrone LN, Meuli GJ, Wang RJ, Maleta KM, et al. Antibiotics as part of the management of severe acute malnutrition. *The New England journal of medicine*. 2013;368(5):425-435.

14. Nahar B, Hossain MI, Hamadani JD, Ahmed T, Huda SN, Grantham-McGregor SM, et al. Effects of a community-based approach of food and psychosocial stimulation on growth and development of severely malnourished children in Bangladesh: a randomised trial. *European journal of clinical nutrition*. 2012;66(6):701-709.
15. Akech SO, Karisa J, Nakamya P, Boga M, Maitland K. Phase II trial of isotonic fluid resuscitation in Kenyan children with severe malnutrition and hypovolaemia. *BMC pediatrics*. 2010;10:71.
16. Kerac M, Bunn J, Seal A, Thindwa M, Tomkins A, Sadler K, et al. Probiotics and prebiotics for severe acute malnutrition (PRONUT study): a double-blind efficacy randomised controlled trial in Malawi. *Lancet*. 2009;374(9684):136-144.
17. Dubray C, Ibrahim SA, Abdelmutalib M, Guerin PJ, Dantoine F, Belanger F, et al. Treatment of severe malnutrition with 2-day intramuscular ceftriaxone vs 5-day amoxicillin. *Ann Trop Paediatr*. 2008;28(1):13-22.
